# Aerosol-Assisted Fast Formulating Uniform Pharmaceutical Polymer Microparticles with Variable Properties toward pH-Sensitive Controlled Drug Release

**DOI:** 10.3390/polym8050195

**Published:** 2016-05-14

**Authors:** Hong Lei, Xingmin Gao, Winston Duo Wu, Zhangxiong Wu, Xiao Dong Chen

**Affiliations:** Suzhou Key Laboratory of Green Chemical Engineering, School of Chemical and Environmental Engineering, College of Chemistry, Chemical Engineering and Materials Science, Soochow University, Suzhou 215123, China; 20134209238@stu.suda.edu.cn (H.L.); 20134209233@stu.suda.edu.cn (X.G.); xdchen@mail.suda.edu.cn (X.D.C.)

**Keywords:** aerosol method, spray drying, polymer microparticle, microencapsulation, drug delivery

## Abstract

Microencapsulation is highly attractive for oral drug delivery. Microparticles are a common form of drug carrier for this purpose. There is still a high demand on efficient methods to fabricate microparticles with uniform sizes and well-controlled particle properties. In this paper, uniform hydroxypropyl methylcellulose phthalate (HPMCP)-based pharmaceutical microparticles loaded with either hydrophobic or hydrophilic model drugs have been directly formulated by using a unique aerosol technique, *i.e.*, the microfluidic spray drying technology. A series of microparticles of controllable particle sizes, shapes, and structures are fabricated by tuning the solvent composition and drying temperature. It is found that a more volatile solvent and a higher drying temperature can result in fast evaporation rates to form microparticles of larger lateral size, more irregular shape, and denser matrix. The nature of the model drugs also plays an important role in determining particle properties. The drug release behaviors of the pharmaceutical microparticles are dependent on their structural properties and the nature of a specific drug, as well as sensitive to the pH value of the release medium. Most importantly, drugs in the microparticles obtained by using a more volatile solvent or a higher drying temperature can be well protected from degradation in harsh simulated gastric fluids due to the dense structures of the microparticles, while they can be fast-released in simulated intestinal fluids through particle dissolution. These pharmaceutical microparticles are potentially useful for site-specific (enteric) delivery of orally-administered drugs.

## 1. Introduction

Microencapsulation is highly important for oral and transdermal drug deliveries [1,2,3,4,5]. In particular, microparticles as drug carriers capable of pH-sensitive controlled release play a vital role in site-selective (such as enteric) delivery of orally-administered drugs for therapeutic applications [6,7,8,9,10,11]. Ideally, the embedded drugs should be well protected by the carrier materials from degradation in the highly-acidic gastric fluid and released in a desirable way while reaching the intestinal tract for efficient absorption [12,13]. Therefore, the selection and control of the structural properties of microparticle carriers are very important [14,15]. Hydroxypropyl methylcellulose phthalate (HPMCP), a generally-recognized-as-safe (GRAS) cellulose derivative, is widely adopted as an enteric polymer for drug delivery due to its pH-dependent solubility, *i.e.*, low solubility in the harsh gastric fluid while the opposite in the intestinal tract [12,16,17]. However, low solubility does not necessarily guarantee drug protection. The structural properties, especially the particle size/uniformity and surface/internal porosity, should also be well controlled to make the encapsulated drugs well protected and to understand release behaviors [2,3,18,19]. Additionally, drug-carrier interactions should be considered for better drug protection and control of particle properties [2].

To fabricate microparticles for the purpose of controlled drug delivery, a series of approaches have been developed, such as the emulsion method [10,20,21,22], microfluidics [20,23], electrostatic droplet method [24,25], membrane filtration [26], supercritical CO_2_ processing [27,28], templating [29], electrohydrodynamic atomization technique [30], and so on. Comparatively, aerosol-based techniques, particularly spray drying technology, is a continuous and low-cost method to manufacture powdered microparticles [31,32,33]. In addition, microparticles prepared through aerosol methods are usually of high chemical utilization efficiency (theoretically 100%) through avoiding washing and purification steps. In aerosol methods, liquids are atomized to droplets, which are subsequently dried and/or thermally treated to obtain targeted particles [34,35,36,37]. During fast solvent evaporation, solutes in liquid droplets are driven away far from equilibrium and forced to be assembled together, leading to unique co-assembly mechanisms and boundary phenomena, and then to novel materials with unique structures, such as mesoporous, core-shell, and hierarchical structures [34,35,36,37,38,39,40,41].

Conventional aerosol techniques, such as conventional spray drying, however, often produce microparticles with relatively small sizes (typically tens of nanometers to a few micrometers) and very broad particle size distributions [34,35,36,37,38,39,40,41,42], mainly due to the atomized droplets of a wide range of size distributions and complex travelling trajectories, which experience different drying histories within the same product batch. Consequently, the effect of a particular process parameter on microparticle properties and a particular property on particle performance cannot be accurately figured out. To tackle such drawbacks, we have recently introduced a specially-designed micro-fluidic-jet spray dryer (MFJSD) that is capable of producing uniform and large-sized microparticles ranging from 5 to 300 µm with controllable characteristics and functionalities [43,44,45]. The properties of spray-dried microparticles are significantly influenced by precursor formulation, such as composition and solvent type, and spray drying conditions, such as air flow rate and drying temperature. Therefore, in order to produce pharmaceutical microparticles capable of pH-sensitive drug release, the structures of the microparticles should be precisely controlled and the correlations between particle structure and drug release behavior should be understood.

Herein in this work, for the first time, we explored the use of MFJSD for the one-step straightforward fabrication and control of structural properties of drug-loaded HPMCP-based microparticles for pH-sensitive drug delivery. Hydrocortisone and lysine were selected as a hydrophobic and a hydrophilic model drug, respectively, to investigate their influences on particle properties and release behaviors. The effects of precursor formulation, including solvent composition and drying temperature, on spray dried particle characteristics and release behaviors have been studied in detail. A series of uniform HPMCP-based pharmaceutical microparticles capable of pH-sensitive drug delivery have been successfully fabricated, and their drug release behaviors have been well correlated with their structural properties.

## 2. Materials and Methods

### 2.1. Chemicals

Hydroxypropyl methylcellulose phthalate (HPMCP) was purchased from Yolne (Shanghai, China). Hydrocortisone of biochemical reagent was purchased from Sinopharm Chemical Reagent Co., Ltd. (Shanghai, China). Lysine was kindly provided by COFCO (Beijing, China). Ethanol, acetone, hydrochloric acid, ammonium hydroxide solution (25~28 wt %), dimethyl sulfoxide, ninhydrin, and lithium hydroxide monohydrate were of analytical grade. Deionized water (Milli-Q) was used throughout the experiments wherever required.

### 2.2. Precursor Preparation

The precursors for spray drying were prepared by directly dispersing HPMCP into certain solvents. To investigate the effects of solvents, different formulations of precursors were prepared (Table S1). The total mass content of excipients was set to be 2.5% (*w*/*v*). A certain amount, typically 2.5 wt %, of a specific model drug was added into the precursor solutions for one-step formulating pharmaceutical microparticles.

### 2.3. Microparticle Fabrication

Briefly, the precursor solutions were fed into a standard steel reservoir; and compressed air was used to force the liquid in the reservoir to jet through the microfluidic aerosol nozzle (MFAN, home-made, Suzhou, China, Figure S1a). The liquid flowing rate was controlled by changing the air pressure. The liquid jet was broken-up into droplets by disturbance from vibrating piezoceramics on the nozzle. The vibration frequency was tuned in order to obtain monodisperse droplets. A new generation of MFJSD-6 (home-made, Suzhou, China, Figure S1b) was used to produce microparticles. The inlet temperature was controlled from 95 to 205 °C for various precursor solutions. The flow rate of the hot air was 250 L/min. The collected microparticles were stored in desiccators for further characterizations and drug release tests.

### 2.4. Particle Characterization

The morphology and structure of microparticles before and after drug release tests were examined by scanning electron microscopy (SEM, S-4700, Hitachi High Technologies Corporation, Tokyo, Japan). Particle size and size distribution were acquired by analyzing SEM images containing over 1000 particles by using Shineso (SHINESO, Hangzhou, China). The average particle size $\bar{d}$ was defined as $\bar{\text{d}}=\sum_{i=1}^{n}d_{i}/N$ and the span of size distribution was described as span = (*d*_90_ − *d*_10_)/*d*_50_, where $d_{i}$ was the diameter of the i-th particle, N was the total number of micropaticles, and *d*_90_, *d*_50_, and *d*_10_ were the cumulative particle sizes at 90%, 50%, and 10%, respectively.

Microparticle density was assessed by filling 0.5~2 g powders into a 5 mL graduated cylinder, and the weight and volume occupied by the powder were recorded. The mass/volume ratio before tapping was calculated as bulk density. The tap density of the powder was then evaluated by tapping the syringe onto a level surface at a height of about 2 cm until no change in volume is observed [46]. Carr’s index was calculated as (tap density-bulk density) × 100/tap density.

The crystalline characteristics of the raw drugs and microparticles were tested by using the powder X-ray diffraction (XRD) technique (X’Pert-Pro MPD, PANalytical B.V., Almelo, Netherlands), with a Cu Kα radiation source and a scan range of 1°/min. The powders were pressed onto quartz blocks by using a glass slide for direct data recording.

### 2.5. In Vitro Drug Release Test

The swelling and dissolution behaviors of typical pharmaceutical microparticles were studied. Briefly, a small amount of microparticles was placed on a glass slide and a drop of PBS (phosphate buffer solution) or simulated gastric juice (0.1 M HCl) at around 25 °C was dropped on the glass surface next to the microparticles. Then, the microparticles contacted with water and were infiltrated slowly. The dissolution processes in PBS and swelling phenomena in simulated gastric juice were recorded using an optical microscope (OLYMPUS-CX31, Olympus Corporation, Tokyo, Japan) and a series of images at different time intervals were captured by AE (Adobe After Effects CS4, Adobe Systems Incorporated, San Jose, CA, USA).

In a typical drug-release experiment, 25 mL of PBS release medium (pH value 7.4) or simulated gastric fluid (0.1 M HCl solution, pH value 1.0) was transferred into a flask, and the drug-loaded microparticles (~50 mg) were added into the flask. The flask was kept in a shaking incubator at 37 °C with constant agitation (65 rpm). At certain time intervals, 1 mL of the release medium was withdrawn periodically from the flask and replaced with the same amount of fresh release medium. The release medium was passed through a 0.45 μm membrane filter (Millipore, Shanghai, China) in order to remove insoluble matters. The concentration of hydrocortisone was directly measured by UV–Vis spectroscopy (Spectramax M5, Molecular Devices, Silicon Valley, CA, USA) at a wavelength of 247 nm. For the determination of lysine concentration, due to its non-obvious absorption peak in the ultraviolet and visible light areas, a ninhydrin method was adopted for lysine quantification [47,48]. Briefly, samples of releasing medium (0.5 mL) and ninhydrin solution (0.5 mL) were put in tubes and heated in a boiling water bath for 10 min. After heating, the tubes were fully cooled in an ice bath. Then, 2.5 mL of 50% alcohol was added into each tube and thoroughly mixed with a vortex mixer for 15 s. Then, 200 µL of the reaction mixture was transferred into quartz 96-well plates and the lysine concentration was determined by measuring the absorbance values at 570 nm.

## 3. Results and Discussion

### 3.1. Particle Formation Process

**Scheme 1:**
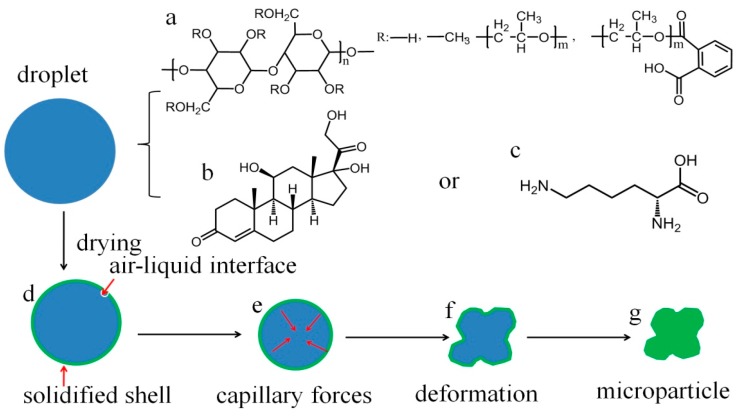
The molecular structures of the HPMCP polymer (**a**); the model drugs hydrocortisone (**b**); lysine (**c**); and a representation of the formation process (**d**–**g**) of the pharmaceutical microparticles via microfluidic spray drying.

**Figure 1:**
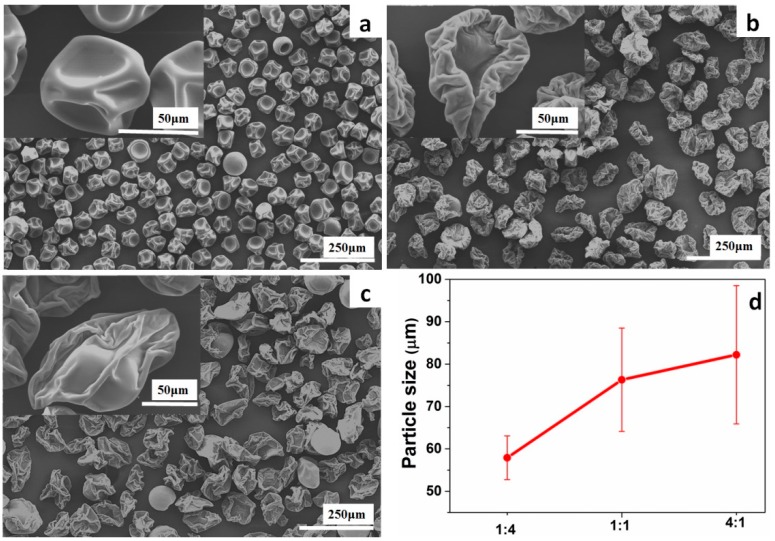
SEM images of the hydrocortisone-loaded microparticles obtained at a drying temperature of 155 °C with an ethanol/water mixed solvent of 1:4 (**a**); 1:1 (**b**); and 4:1 (**c**) volume ratio, respectively; and the particle size variation trend (**d**) with increasing ethanol/water volume ratio.

Solutions composed of HPMCP (Scheme 1a) as the carrier material, hydrophobic hydrocortisone (Scheme 1b) or hydrophilic lysine (Scheme 1c) as the model drug, and ethanol/water mixture as the solvent, were atomized into uniform droplets and directly assembled into uniform microparticles (Scheme 1d–g) via spray drying. At a solid content of 2.5% (*w*/*v*), a hydrocortisone-to-HPMCP mass ratio of 0.025:1, and a drying temperature of 155 °C, uniform, discrete, and dimpled spherical microparticles of ~57.9 µm in size (Figure 1a) were obtained by using an ethanol/water mixture of 1:4 volume ratio as the solvent. The surfaces of these microparticles are smooth and dense (Figure 1a inset), indicating the drug molecules are encapsulated inside the HPMCP matrix. The deformation of spherical droplets into dimpled microparticles originates from surface tension gradients of drying droplets. In general, the droplet air/liquid interface recedes while drying (Scheme 1d), and is gradually concentrated by the precursor ingredients that diffuse inward according to Fick’s law [49].When the ingredients reach saturation, the droplet surface will be solidified to form a shell and stop shrinking (Scheme 1d,e). On the other hand, drying induces temperature gradients (interior < interface temperature), making surface tension differences (interior > external), and preferential evaporation of ethanol leaves behind more water, further enlarging the surface tension differences, thus creating inward capillary forces (Scheme 1e). The soft nature of the HPMCP polymer (with remaining water solvent) makes the shell unable to withstand the capillary forces, leading to shape deformation (Scheme 1f) and subsequently dimpled or crumpled microparticles (Scheme 1g).

### 3.2. Solvent Effect on Particle Property

With other parameters unchanged, by increasing the ethanol/water volume ratio from 1:4 to 1:1, and 4:1, severely crumpled microparticles of more irregular shapes, highly-wrinkled surfaces, and increasing lateral particle sizes (~76.3 and 82.2 µm) were obtained (Figure 1b–d). This solvent effect can be ascribed to the increased evaporation rate by replacing water with more ethanol of higher vapor pressure. With faster solvent removal, the particle shell may form earlier and stabilize the drying droplets, thus leading to increased particle sizes. The fast evaporation rate may also trigger longer paths of receding air/liquid interface and temperature gradient; thus, the capillary forces can drive the drying droplets to crumple more and the surfaces to wrinkle more as well. The solvent effect was further validated by using an acetone/water mixture of 9:1 volume ratio as the solvent. The more volatile acetone solvent further accelerated evaporation, leading to larger-sized and more irregular microparticles, and puffed spherical microparticles of even larger sizes (Figure S2). Under this condition, the solvent evaporation rate is so fast that all of the solvent in a droplet could be evaporated instantaneously to form a relatively rigid shell with negligible remaining water solvent inside, leading to puffed microparticles with smaller degrees of crumpling and wrinkling.

### 3.3. Drying Temperature Effect on Particle Property

**Figure 2:**
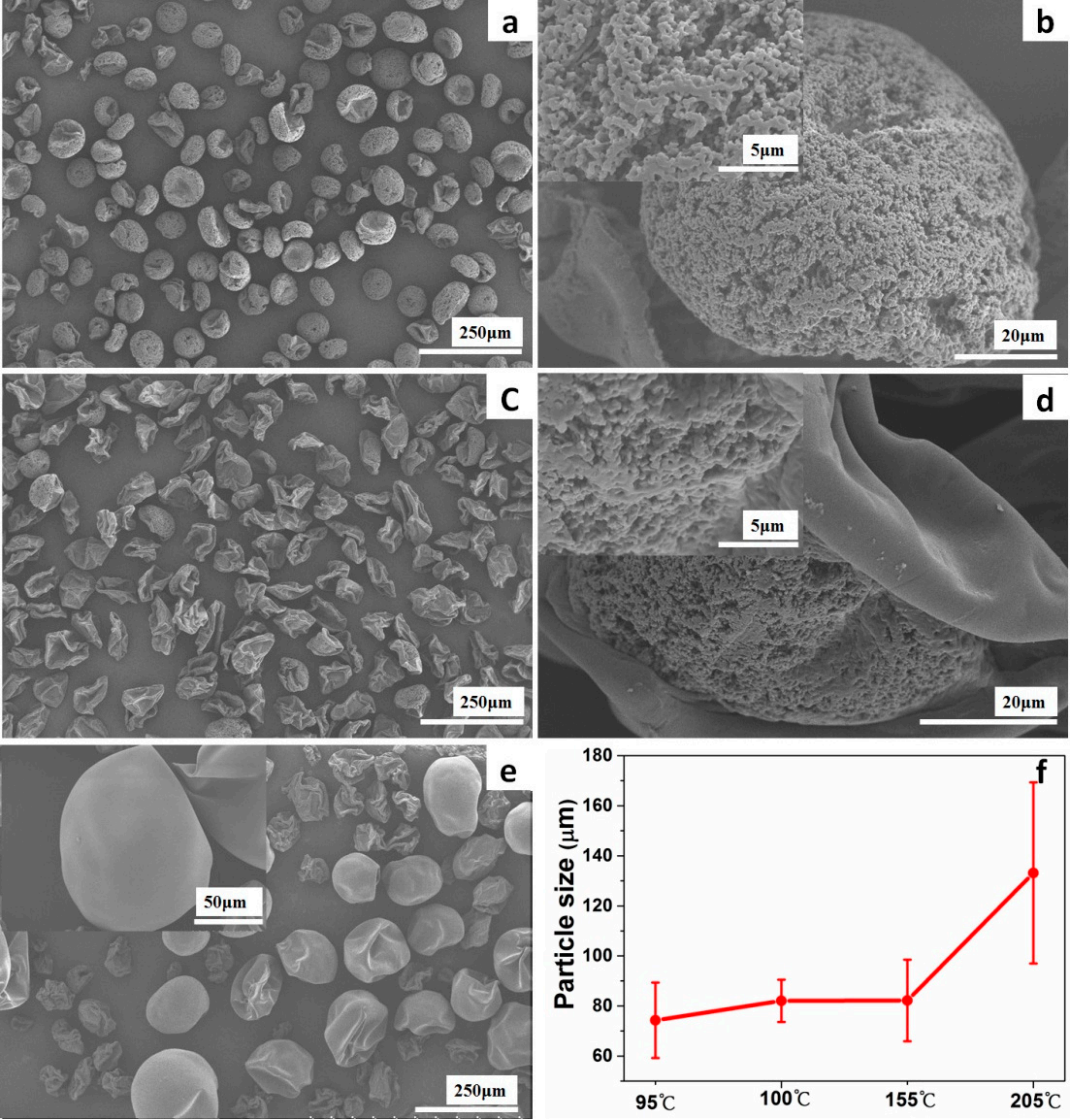
SEM images of the hydrocortisone-loaded microparticles obtained with an ethanol/water mixed solvent of 4:1 volume ratio at a drying temperature of 95 (**a**,**b**); 100 (**c**,**d**); and 205 °C (**e**), respectively; and the particle size variation trend (**f**) with increasing drying temperature.

Drug-loaded microparticles were also formulated at different temperatures to further control particle properties. With a constant solid content of 2.5% (*w*/*v*), a hydrocortisone-to-HPMCP mass ratio of 0.025:1, and an ethanol/water mixture of 4:1 volume ratio as the solvent, uniform and highly open porous microparticles of ~74.3 µm were obtained at a drying temperature of 95 °C (Figure 2a,b). The microparticles are crumpled in single directions, making them bowl-like without surface wrinkles (Figure 2a,b).The average pore size was estimated to be ~200 nm (Figure 2b and inset), which may accelerate drug release. With the drying temperature slightly increased to 100 °C, the resultant particle morphology changed significantly to severely crumpled and surface-wrinkled microparticles of increased lateral sizes (Figure 2c,f). Interestingly, the microparticles are Janus-like with one half porous, while the other half is non-porous (Figure 2d). With the temperature increased to 155 °C, the obtained microparticles turned to totally non-porous with no obvious particle size increase (Figure 1c and Figure 2f). A further increase of the drying temperature to 205 °C led to puffed microparticles of spherical and smooth shape, as well as an abrupt increase in lateral particle size up to ~133 µm (Figure 2e,f). Very similar trends of temperature-dependent particle properties were also observed for the lysine-loaded microparticles (Figure S3). Similar to the solvent effect, the reason for the particle size increase and higher degree of crumpling/wrinkling with increase of drying temperature is the fastened drying rates at higher temperatures. In addition, at a high drying temperature of 205 °C, the skin formed on the semi-dried droplet may experience higher temperature than the melting point of the HPMCP (~145 °C) for a short time when it travelled along the lower part of the dryer, likely resulting in more smoothness of the particle surface. On the other hand, at a low drying temperature of 95 °C, the slow drying rate allows sufficient solute migration and growth due to the existence of remaining water solvent, thus leading to the formation of bowl-like microparticles with open macropores (Figure 2a,b).

### 3.4. Effect of Drug Molecule on Particle Properties

Under the same synthetic conditions, the lysine- and hydrocortisone-encapsulated microparticles show very similar properties and variations with solvent composition and drying temperature (Figure 2 and Figure S3). Two differences were observed, though. Firstly, under the same synthetic conditions, the lysine-encapsulated microparticles present a relatively larger particle size than those of the hydrocortisone-encapsulated counterparts (Table S2, for example, comparing the size differences of the hydrocortisone- and lysine-loaded microparticles obtained at 100 or 155 °C). This is probably because of the existence of stronger molecular interactions between lysine (with amino groups, Scheme 1c) and the HPMCP matrix (with carboxylic groups, Scheme 1a), which can accelerate particle solidification during drying. This assumption is verified by the fact that the lateral particle size increases from ~94.6 to 108.8 µm with the increase of the lysine-to-HPMCP mass ratio from 0.025 to 0.1 (Figure S3b,e and Table S2). Secondly, open macroporous bowl-like hydrocortisone-loaded microparticles can be obtained only at a low temperature of 95 °C (Figure 2a), while lysine-loaded microparticles with identical properties can be formed at 100 °C (Figure S3a). This is probably because the hydrophilic lysine molecules can help sustain the water solvent to allow sufficient solute aggregation and growth to form macropores even at relatively higher temperatures.

### 3.5. Particle Porosity and Density

**Figure 3:**
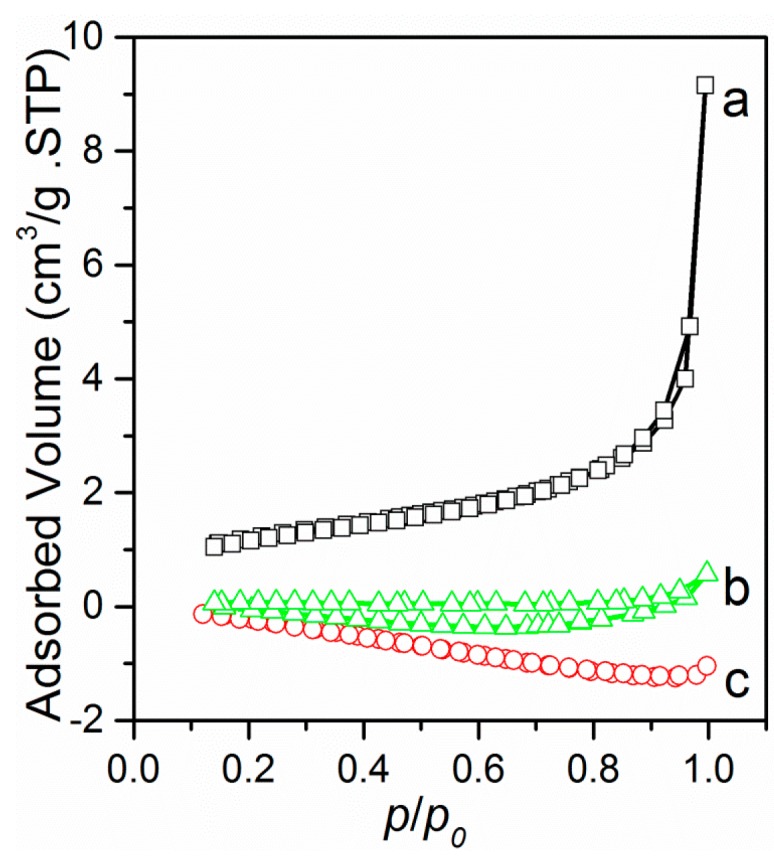
N_2_ sorption isotherms of the lysine-loaded microparticles obtained with an ethanol/water solvent of 4:1 volume ratio at a drying temperature of 100 (**a**); 155 (**b**); and 205 °C (**c**), respectively.

The porous nature of the bowl-like microparticles was verified by the high surface area (~5.4 m^2^/g) of the sample (Figure 3a). With the drying temperature increased to 155 °C or higher, no nitrogen porosity was detected for both the hydrocortisone- and lysine-encapsulated microparticles (Figure 3b,c). At high drying temperature the surface of droplet/particle may be molten, likely leading to a decrease in porosity. There is also a trend that the surface area of the microparticles obtained at the same temperature decreases with the increasing ethanol-to-water volume ratio of the solvents (Figure S4), in accordance with the fact that higher ethanol-to-water volume ratio leads to faster evaporation rates and solidification of particle shells.

The flowing property of pharmaceutical microparticles is important for dosing efficiency and product handling, such as mixing, packaging, storage, *etc*. Carr’s index (CI) of less than 25% usually indicates fluid powder, which means that the closer bulk and tap densities are, the better the powder flowability. The skeletal density of HPMCP polymer is ~1.25 cm^3^/g, while both the bulk and tap densities of the hydrocortisone- and lysine-encapsulated microparticles are far smaller than this value due to the intraparticle porosity and/or inter-particle voids (Table S2). Moreover, both the bulk and tap densities of the obtained microparticles significantly declined with precursor solvent composed of more ethanol, as well as with increased drying temperature (Table S2), much likely resulting from the larger particle size and more irregular shape. The CIs for the hydrocortisone-encapsulated microparticles obtained from different solvent compositions are similar and close to 25%, a sign of good flowability. For the lysine-encapsulated microparticles, the flowability is better for the microparticles obtained at 100 °C than those obtained at higher drying temperatures, probably due to larger and more irregular particle sizes hindering particle compacting.

### 3.6. States of Drugs in the Microparticles

**Figure 4:**
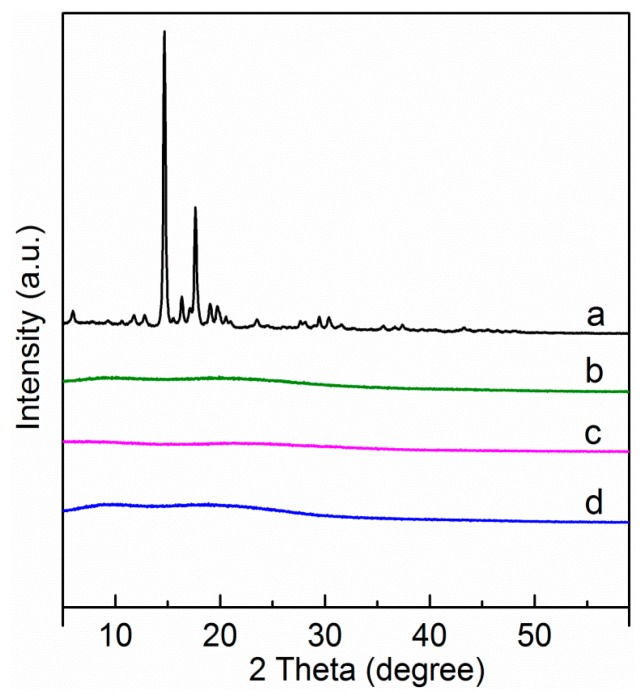
XRD patterns of the raw hydrocortisone drug (**a**); and the hydrocortisone-loaded microparticles obtained at a drying temperature of 155 °C with an ethanol/water solvent of 1:4 (**b**); 1:1 (**c**); and 4:1 (**d**) volume ratio, respectively.

While the raw drugs are highly crystalline (Figure 4a and Figure S5a), both the encapsulated hydrocortisone and lysine drugs in the microparticles are amorphous regardless of the precursor solvent composition and drying temperature (Figure 4b–d and Figure S5b–d), indicating the drugs are well dispersed in the HCMCP matrix. During the fast spray drying process, the drug molecules are driven far away from equilibrium growth conditions. In addition, the drug molecules could interact strongly with the HPMCP matrix. Therefore, growth and molecular organization of drug molecules are limited, leading to well-dispersed amorphous drug particles. This is believed to be an advantage in terms of facilitating fast drug dissolution and absorption [50].

### 3.7. Drug Release Behaviors

**Figure 5:**
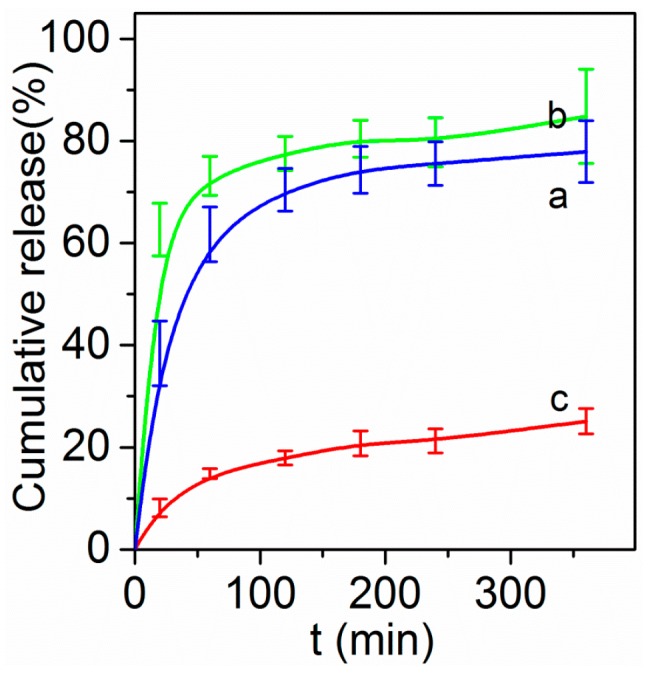
Cumulative release of hydrocortisone drug in a simulated gastric fluid from the microparticles obtained at a drying temperature of 155 °C with an ethanol/water mixed solvent of 1:4 (**a**); 1:1 (**b**); and 4:1 (**c**) volume ratio, respectively.

The release behaviors of the two model drugs were found to be dependent on the structures of the microparticles, as well as sensitive to the pH value of the release environment. In simulated gastric solutions (0.1 M HCl solution), at the same drying temperature (155 °C), for the microparticles obtained at low ethanol/water ratios (1:4 and 1:1), the hydrocortisone drug were released fast with an initial burst of ~70% within one hour (Figure 5a,b). Afterwards, the drug was released slowly with a total of ~80% released up to 6 h (Figure 5a,b), indicating the drugs cannot be well protected in these microparticles against the gastric environment. On the contrary, for the microparticles obtained at a high ethanol/water ratios (4:1), the drug was released very slowly from the beginning (Figure 5c), with only ~20% released within up to 6 h, indicating the drugs in these microparticles can be well protected against the harsh gastric environment.

**Figure 6:**
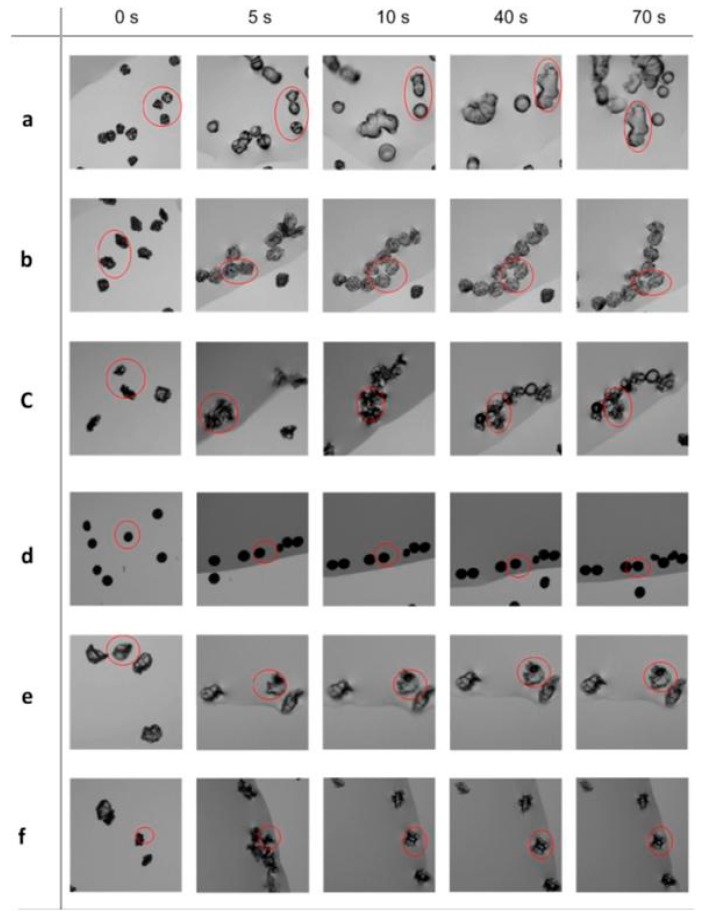
Time-lapse tracking photographs of swelling behaviors in simulate gastric solutions of the microparticles obtained at a drying temperature of 155 °C with an ethanol/water mixed solvent of 1:4 (**a**); 1:1 (**b**); and 4:1 (**c**); volume ratio, and those obtained with an ethanol/water solvent of 4:1 volume ratio at a drying temperature of 95 (**d**); 100 (**e**); and 205 °C (**f**), respectively. A set of the same microparticles in each row was enclosed in red cycles to clearly show their swelling changes with time.

The above different release behaviors are directly associated with the different structures of the microparticles. The higher ethanol fraction (4:1) in the precursor solvent leads to a faster drying rate, and subsequently to a larger lateral particle size with denser particle shells (Figure 1). As a result, the access of water molecules from the simulated gastric solution into the interior of the microparticles is severely retarded, and the drug molecules are released only very slowly. Tracking of the swelling behaviors of the microparticles in simulated gastric solution further verifies the different release properties. For the microparticles obtained from solvents with low ethanol/water ratios (1:4, 1:1), fast particle swelling and inter-particle mergence were observed (Figure 6a,b), which could trigger fast drug release. On the contrary, for the microparticles obtained from a solvent with the high ethanol/water ratio (4:1), due to the more dense particle shells, only slow and small-degree particle swelling was observed (Figure 6c), which could retard drug release.

**Figure 7:**
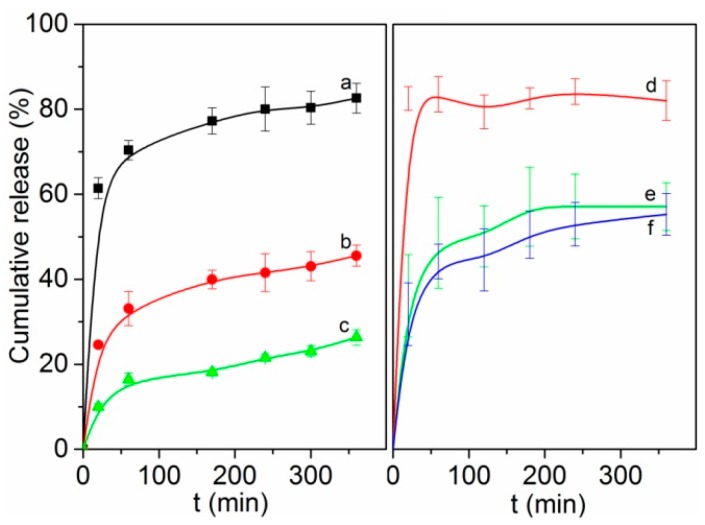
Cumulative release of hydrocortisone (**a**–**c**); and lysine (**d**–**f**) in simulated gastric fluids from the microparticles obtained with an ethanol/water mixed solvent of 4:1 volume ratio at a drying temperature of 95 (**a**); 100 (**b**,**d**); 155 (**c**,**e**); and 205 °C (**f**), respectively.

Microparticles obtained at different drying temperatures also show significantly different drug release behaviors. With the same solvent composition (ethanol/water mixture of 4:1 volume ratio), for the microparticles obtained at 95 °C, the encapsulated hydrocortisone drug was released fast with an initial burst of 60%, followed by a gradual release up to ~80% within 6 h (Figure 7a). For those obtained at 100 °C, the release rate became much slower, with an initial release of 25% at 20 min and then a gradual one up to ~40% at 6 h (Figure 7b). Notably, a much slower rate was present for those obtained at a higher temperature of 155 °C (Figure 7c). For the lysine-encapsulated microparticles, very similar temperature-dependent releasing trends were observed (Figure 7d–f). The above different drying-temperature-induced release behaviors can also be well correlated with the structural properties of the corresponding microparticles. At low drying temperatures, the obtained microparticles are open porous (Figure 2a and Figure S3a) so that the access of water into the interior of these microparticles is facilitated, and the drug molecules inside can be released very fast. Tracking of these microparticles reveals that they were not obviously swollen in the simulated gastric solution (Figure 6d), further indicating the drug release mainly relies on direct mass transportation through the macropores. On the contrary, the microparticles obtained at higher temperatures (155 °C or higher) are quite dense (Figure 1) without any detectable N_2_ porosity (Figure 3). Besides, at high drying temperatures, the obtained microparticles appear crumpled/wrinkled or puffed morphology with a possibly molten surface, which could restrict drug release. Therefore, mass exchange between outside and internal microparticles is much retarded, leading to much slower drug release rates. Tracking of these microparticles reveals that the microparticles were swollen in the simulated gastric solution (Figure 6e,f), indicating the drug release mainly relies on slow mass exchange.

Interestingly, under the same synthetic conditions, the lysine-loaded microparticles show faster drug release rates as compared to those of the hydrocortisone-loaded counterparts (Figure 7). For example, for the microparticles obtained at 100 °C, the released amount of lysine was up to 80% within 1 h and without noticeable release afterwards (Figure 7d). However, the released amount of hydrocortisone was only ~32 wt % within 1 h and with a further slow release stage (Figure 7b). The reason is mainly due to the difference in surface pore structure. The former shows highly open porous structure (Figure S3a) while the latter is only half-particle porous and the pores are less open (Figure 2c,d). Similar release difference was also found in the microparticles obtained at 155 °C (Figure 7c,e). At this temperature, both the lysine- and hydrocortisone-loaded microparticles are quite dense; therefore, the faster release of lysine is probably due to its more basic and hydrophilic nature and smaller molecular size as compared with hydrocortisone, which make it more easily to be dissolved in the simulated gastric solution. This is verified by the much faster release rate of the lysine-loaded microparticles obtained at 100 °C (Figure 7d) than that of the hydrocortisone-loaded microparticles obtained at 95 °C (Figure 7a), while both types of microparticles show highly open porous structures (Figure 2a and Figure S3a).

After the release tests in simulated gastric solutions, both the hydrocortisone- and lysine-loaded microparticles show no obvious changes in particle morphology and structure (Figures S6 and S7). These results indicate the microparticles can be well preserved in the harsh simulated gastric environments.

**Figure 8:**
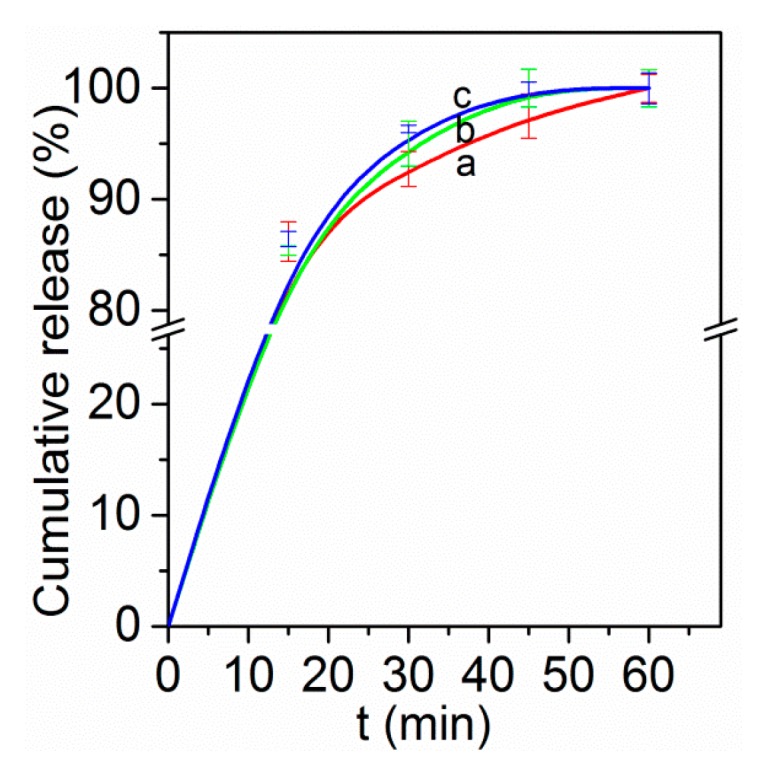
Cumulative release of lysine in PBS solutions from the microparticles obtained with an ethanol/water solvent of 4:1 volume ratio at a drying temperature of 100 (**a**); 155 (**b**); and 205 °C (**c**), respectively.

**Figure 9:**
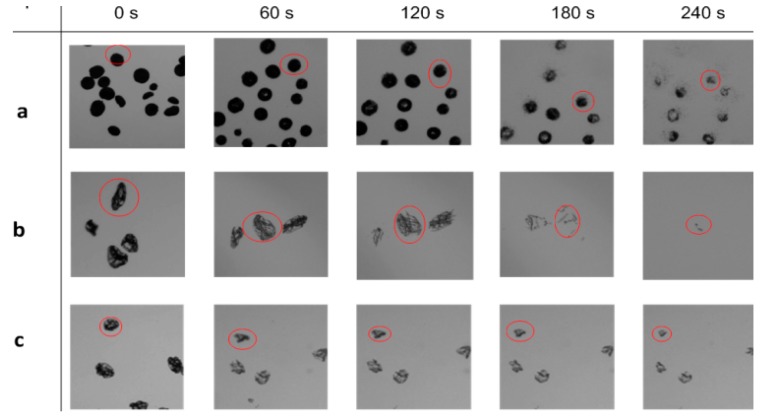
Time-lapse tracking photographs of dissolution behaviors in PBS solutions of the lysine-loaded microparticles obtained with an ethanol/water solvent of 4:1 volume ratio at a drying temperature of 100 (**a**); 155 (**b**); and 205 °C (**c**), respectively.

The drug release is pH-sensitive with totally different release behaviors near a neutral environment. To mimic the enteric environment, a PBS solution (pH value 7.4) was used as the release medium. Notably, ~90% drug molecules (either lysine or hydrocortisone) were released in the first 30 min and almost 100% released within 1 h for all the drug-encapsulated microparticles regardless of their structural properties (Figure 8). Track of the microparticles in PBS solution indicates that the release mechanism lies in the fast dissolution of the whole microparticles in PBS solution (Figure 9 and Figure S8), in accordance with literature that HPMCP is a polymer with high solubility in the intestinal tract [51,52]. The above results indicate that site-specific drug delivery could be achieved by controlling the microparticle properties.

## 4. Conclusions

This paper has demonstrated the use of microfluidic jet spray drying to directly fabricate drug-encapsulated HPMCP polymer microparticles with uniform and tunable sizes (57~133 µm), variable morphologies (bowl-like, dimpled, crumpled, and spherical) and structures (porous, Janus-like, porous, and dense). Adoption of either more volatile solvents or higher drying temperatures leads to microparticles with larger lateral particle sizes, more severely crumpled morphologies and wrinkled surfaces, and declined porosity. At high drying temperature the surface of droplet/particle may be molten during spray drying process, also likely leading to more smoothness of the particle surface and decrease in porosity. As compared with the hydrophobic hydrocortisone, the hydrophilic and basic lysine model drug tends to interact more strongly with HMPCP leading to relatively larger particle sizes, to sustain more water leading to the formation of open macropores at a relatively higher drying temperature, and to be released relatively faster. The release behaviors are particle-property dependant and pH-sensitive. Drugs in the microparticles obtained at a drying temperature of 155 °C or higher, and those obtained by using a solvent with a high ethanol/water ratio can be well protected in simulated gastric solutions and subsequently be fast released in PBS solutions. Therefore, these microparticles are capable of protecting drugs against harsh gastric fluids and releasing them in enteric fluids. The current method could provide a robust route for the fabrication for pharmaceutical microparticles for site-specific delivery of orally administrated drugs of different molecular properties.

## Supplementary Materials

Supplementary Materials can be found at www.mdpi.com/2073-4360/8/5/195/s1. Figure S1: Photographs of the MFAN nozzle (a); and the spray drying set-up (b); Figure S2: SEM images of the hydrocortisone-loaded microparticles obtained by using an acetone/water solvent of 9:1 volume ratio at a drying temperature of 155 °C; Figure S3: SEM images of the lysine-loaded microparticles obtained with an ethanol/water mixed solvent of 4:1 volume ratio at a drying temperature of 100 (a); 155 (b); and 205 °C (c); respectively, and the particle size variation trend (d) with increasing drying temperature; (e) and (f) are the SEM images of the lysine-loaded microparticles with 10% drug loading obtained with an ethanol/water mixed solvent of 4:1 volume ratio at a drying temperature o155 °C; Figure S4: N_2_ sorption isotherms of the lysine-loaded microparticles obtained with an ethanol/water solvent of 1: 4 (a); and 1:1 (b) volume ratio at a drying temperature of 155 °C; Figure S5: XRD patterns of the raw lysine drug (a); and the lysine-loaded microparticles obtained with an ethanol/water mixed solvent of 4:1 at a drying temperature of 100 (b); 155 (c); and 205 °C (d), respectively; Figure S6: After the drug release tests in simulated gastric solutions, SEM images of the hydrocortisone-loaded microparticles obtained at a drying temperature of 155 °C with an ethanol/water mixed solvent of 1:4 (a); 1:1 (b); and 4:1 (c) volume ratio, respectively; Figure S7: After the drug release tests in simulated gastric solutions, SEM images of the lysine-loaded microparticles obtained with an ethanol/water mixed solvent of a volume ratio of 4:1 at a drying temperature of 100 (a); 155 (b); and 205 °C (c), respectively; Figure S8: Time-lapse tracking photographs of dissolution behaviors in PBS solutions of the hydrocortisone-loaded microparticles obtained with an ethanol/water mixed solvent of 1:4 (a); 1:1 (b); and 4:1 (c) volume ratio at a drying temperature of 155 °C. Table S1: Summary of the formulations of precursors for the fabrication of a series of pharmaceutical microparticles via microfluidic jet spray drying; Table S2: Data summary of the particle size, bulk density, tap density, and Carr’s Index for the obtained pharmaceutical microparticles.

## Abbreviations

The following abbreviations are used in this manuscript:

HPMCP	Hydroxypropyl methylcellulose phthalate
GRAS	Generally recognized as safe
MFJSD	Micro-fluidic-jet spray dryer
MFAN	Micro-fluidic-aerosol-nozzle
AE	Adobe after effects CS4
CI	Carr’s index
XRD	X-ray diffraction
SEM	Scanning electron microscopy
PBS	Phosphate buffer saline
UV	Ultraviolet
