# Supplementary material for: Aerosol-Assisted Fast Formulating Uniform Pharmaceutical Polymer Microparticles with Variable Properties toward pH-Sensitive Controlled Drug Release
Source: Polymers (Basel). 2016 May 14;8(5):195. doi: 10.3390/polym8050195 (PMC6432404; doi:10.3390/polym8050195)
Supplement: Supplementary file 1 [file polymers-08-00195-s001.pdf]

Supplementary Materials: Aerosol-Assisted Fast Formulating Uniform Pharmaceutical Polymer Microparticles with Variable Properties toward pH-Sensitive Controlled Drug Release

Hong Lei, Xingmin Gao, Winston Duo Wu, Zhangxiong Wu and Xiao Dong Chen

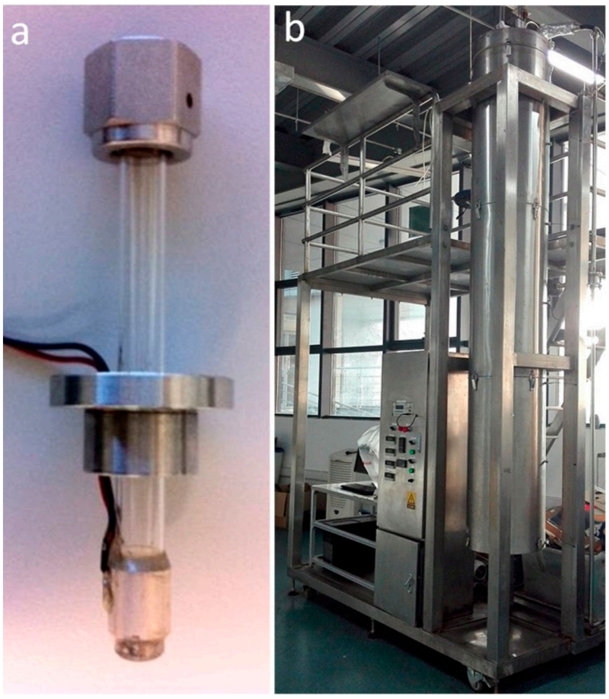

Figure S1. Photographs of the MFAN nozzle (**a**); and the spray drying set-up (**b**).

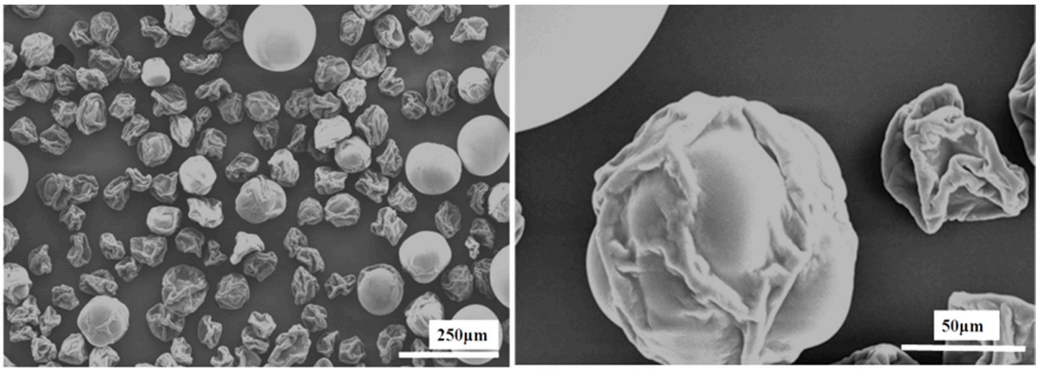

Figure S2. SEM images of the hydrocortisone-loaded microparticles obtained by using an acetone/water solvent of 9:1 volume ratio at a drying temperature of 155 °C.

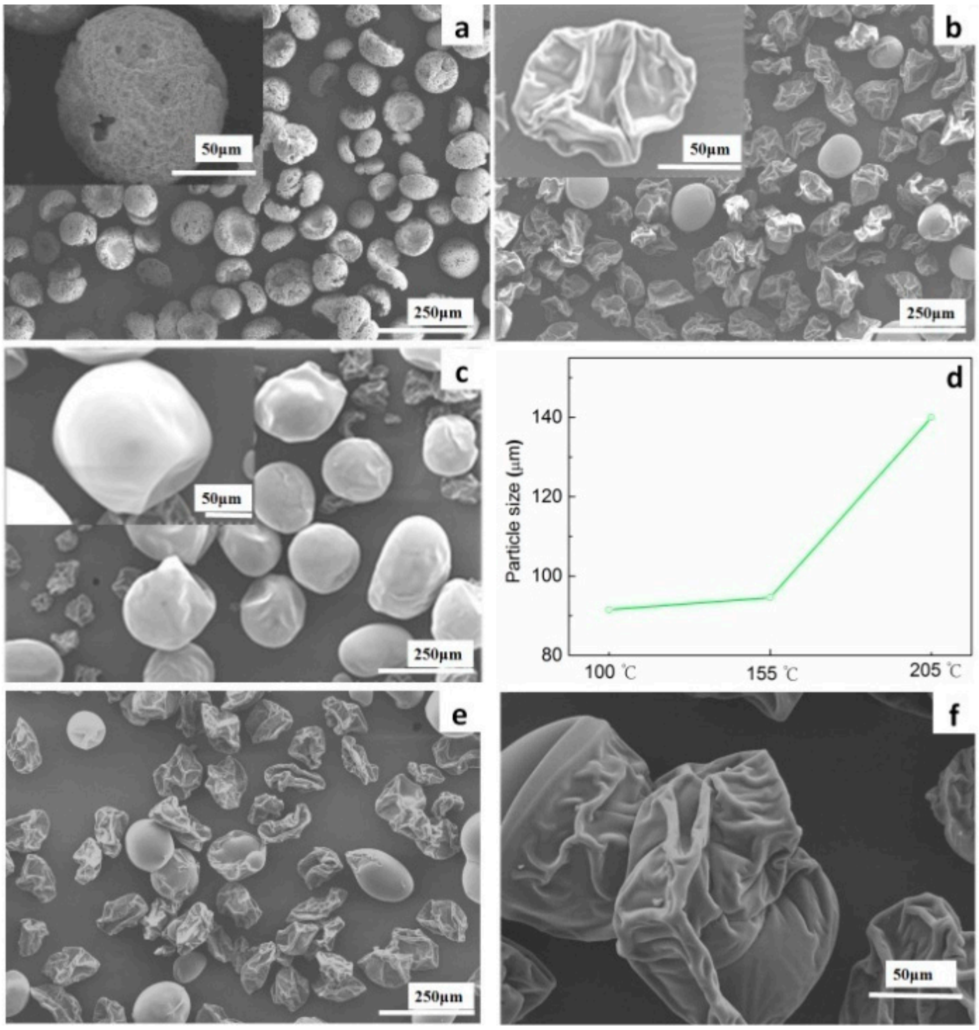

Figure S3. SEM images of the lysine-loaded microparticles obtained with an ethanol/water mixed solvent of 4:1 volume ratio at a drying temperature of 100 (**a**); 155 (**b**); and 205 °C (**c**); respectively, and the particle size variation trend (**d**) with increasing drying temperature; (**e**) and (**f**) are the SEM images of the lysine-loaded microparticles with 10% drug loading obtained with an ethanol/water mixed solvent of 4:1 volume ratio at a drying temperature of 155 °C.

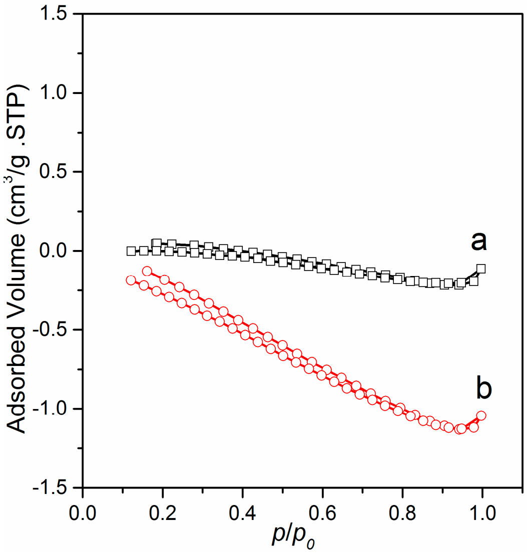

Figure S4. N_2 sorption isotherms of the lysine-loaded microparticles obtained with an ethanol/water solvent of 1:4 (**a**); and 1:1 (**b**) volume ratio at a drying temperature of 155 °C.

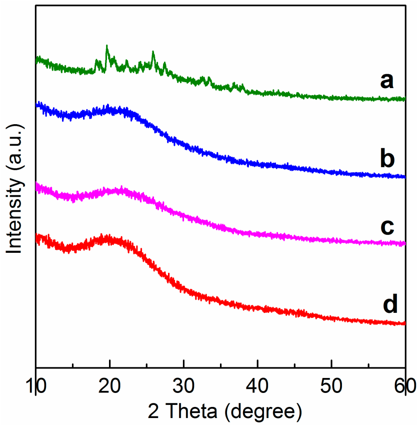

Figure S5. XRD patterns of the raw lysine drug (**a**); and the lysine-loaded microparticles obtained with an ethanol/water mixed solvent of 4:1 at a drying temperature of 100 (**b**); 155 (**c**); and 205 °C (**d**), respectively.

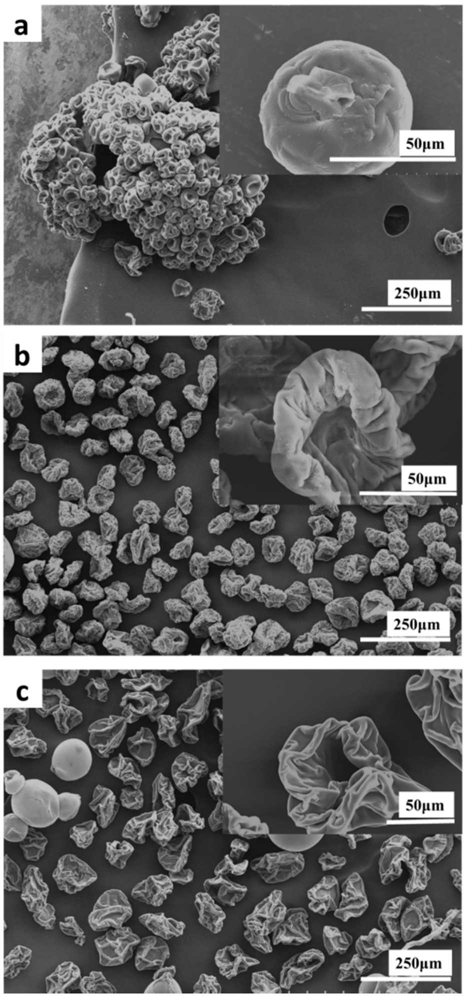

Figure S6. After the drug release tests in simulated gastric solutions, SEM images of the hydrocortisone-loaded microparticles obtained at a drying temperature of 155 °C with an ethanol/water mixed solvent of 1:4 (**a**); 1:1 (**b**); and 4:1 (**c**) volume ratio, respectively.

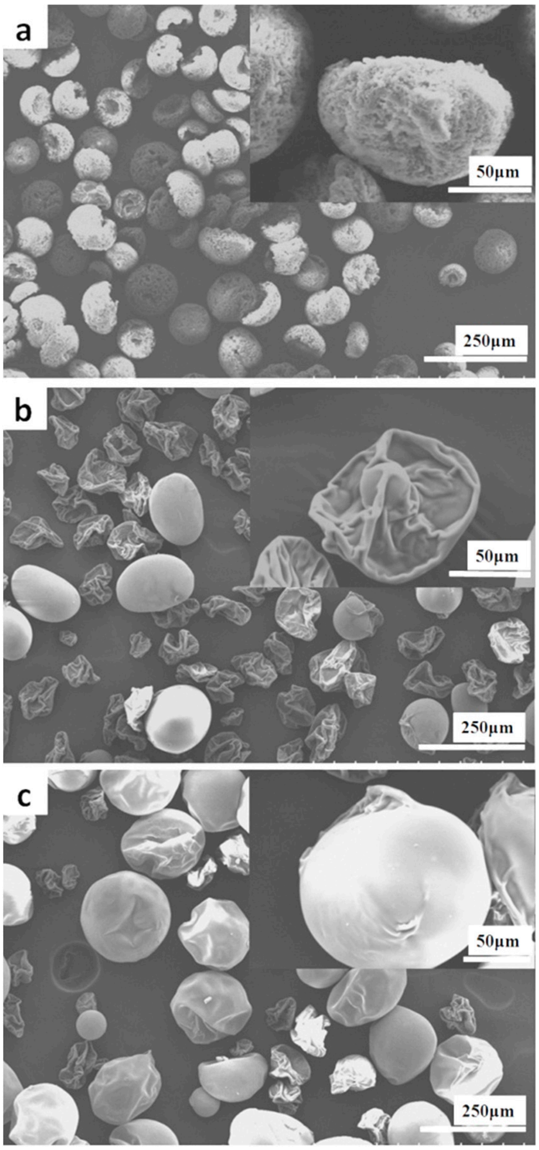

Figure S7. After the drug release tests in simulated gastric solutions, SEM images of the lysine-loaded microparticles obtained with an ethanol/water mixed solvent of a volume ratio of 4:1 at a drying temperature of 100 (**a**); 155 (**b**);and 205 °C (**c**), respectively.

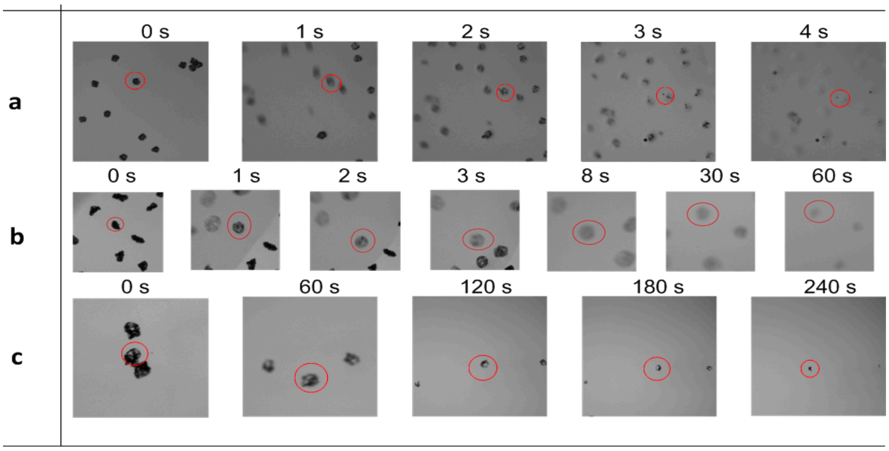

Figure S8. Time-lapse tracking photographs of dissolution behaviors in PBS solutions of the hydrocortisone-loaded microparticles obtained with an ethanol/water mixed solvent of 1:4 (**a**); 1:1 (**b**); and 4:1 (**c**) volume ratio at a drying temperature of 155 °C. A set of the same microparticles in each row was enclosed in red cycles to clearly show their swelling changes with time.

Table S1. Summary of the formulations of precursors for the fabrication of a series of pharmaceutical microparticles via microfluidic jet spray drying.

Sample	Compound			Solvent composition [c]	NH_4OH [d] (*v*/*v*)
	HPMCP [a] (*w*/*v*)	HY(*w*/*w*) [b]	LY(*w*/*w*) [b]		
$H_1HY_{0.025}$-(W_4E_1) [e]	2.5%	0.025	–	W:E = 4:1	0.005
$H_1HY_{0.025}$-(W_1E_1)	2.5%	0.025	–	W:E = 1:1	0.0025
$H_1HY_{0.025}$-(W_1E_4)	2.5%	0.025	–	W:E = 1:4	0
$H_1HY_{0.025}$-(W_1A_9)	2.5%	0.025	–	W:A = 1:9	0
$H_1LY_{0.025}$-(W_1E_4)	2.5%	–	0.025	W:E = 1:4	0
$H_1LY_{0.1}$-(W_1E_4)	2.5%	–	0.1	W:E = 1:4	0

[a] The total solid content of the precursors, e.g., 2.5 g of HPMCP and drug was added into 100 mL solution to obtain a 2.5% precursor solution; [b] the mass ratio of drug to the HPMCP polymer; [c] W, A, and E were short for water, acetone, and ethanol, all ratios were expressed in volume ratios; [d] the volume fraction of ammonia solution (25~28 wt %) to the total volume of the precursor solution. Due to the low solubility of HPMCP in water, ammonia solution was added to help dissolution of HPMCP polymer in the cases of high water/ethanol ratios; [e] in the sample names, H, HY, and LY stand for HPMCP, hydrocortisone, lysine, respectively with the subscript numbers stand for their ratios.

Table S2. Data summary of the particle size, bulk density, tap density, and Carr's Index for the obtained pharmaceutical microparticles.

Sample [a]	Particle size (μm)	Bulk density (g/mL)	Tap density (g/mL)	Carr's index (%)
$H_1HY_{0.025}$-(W_4E_1)-155	57.91 ± 5.14	0.4136	0.5893	29.81
$H_1HY_{0.025}$-(W_1E_1)-155	76.31 ± 12.18	0.3255	0.4367	25.46
$H_1HY_{0.025}$-(W_1E_4)-155	82.20 ± 16.31	0.1506	0.2089	27.92
$H_1HY_{0.025}$-(W_1A_9)-155	83.6 ± 28.73	0.1789	0.2516	28.90
$H_1HY_{0.025}$-(W_1E_4)-95	74.28 ± 15.08	0.2905	0.3925	25.97
$H_1HY_{0.025}$-(W1E4)-100	82.07 ± 8.44	0.2111	0.2967	28.78
$H_1HY_{0.025}$-(W_1E_4)-205	133.12 ± 36.8	0.1076	0.1540	30.11
$H_1LY_{0.025}$-(W_1E_4)-100	91.55 ± 19.4	0.22	0.28	21.43
$H_1LY_{0.025}$-(W_1E_4)-155	94.6 ± 16.5	0.13	0.23	43.47
$H_1LY_{0.025}$-(W_1E_4)-205	140 ± 41.75	0.074	0.11	32.72
$H_1LY_{0.1}$-(W_1E_4)-155	108.8 ± 24.5	0.1639	0.2354	30.37

[a] In the sample names, H, HY, LY, W, E, and A stand for HPMCP, hydrocortisone, lysine, water, ethanol and acetone, respectively. The subscript numbers stand for their ratios.

© 2016 by the authors; licensee MDPI, Basel, Switzerland. This article is an open access article distributed under the terms and conditions of the Creative Commons Attribution (CC-BY) license (http://creativecommons.org/licenses/by/4.0/).
